# Intra-Specific Venom Variation in the Australian Coastal Taipan *Oxyuranus scutellatus*

**DOI:** 10.3390/toxins12080485

**Published:** 2020-07-30

**Authors:** Theo Tasoulis, Anjana Silva, Punnam Chander Veerati, Mark Baker, Wayne C. Hodgson, Nathan Dunstan, Geoffrey K. Isbister

**Affiliations:** 1 Clinical Toxicology Research Group, University of Newcastle, Newcastle, NSW 2308, Australia; punnam.veerati@newcastle.edu.au (P.C.V.); geoff.isbister@gmail.com (G.K.I.); 2 Monash Venom Group, Monash University, Clayton, VIC 3800, Australia; nkanjanasilva@gmail.com (A.S.); wayne.hodgson@monash.edu (W.C.H.); 3 Faculty of Medicine and Allied Sciences, Rajarata University, Anuradhapura-Rambewa Hwy, Anuradhapura 50008, Sri Lanka; 4 Priority Research Centre in Reproductive Biology, University of Newcastle, Newcastle, NSW 2308, Australia; mark.baker@newcastle.edu.au; 5 Venom Supplies, Tanunda, SA 5352, Australia; venoms@venomsupplies.com

**Keywords:** snake venom, taipan, intra-specific variation

## Abstract

Intra-specific venom variation has the potential to provide important insights into the evolution of snake venom, but remains a relatively neglected aspect of snake venom studies. We investigated the venom from 13 individual coastal taipans *Oxyuranus scutellatus* from four localities on the north-east coast of Australia, spanning a distance of 2000 km. The intra-specific variation in taipan venom was considerably less than the inter-specific variation between it and the other Australian elapids to which it was compared. The electrophoretic venom profile of *O. scutellatus* was visually different to six other genera of Australian elapids, but not to its congener inland taipan *O. microlepidotus.* There was minimal geographical variation in taipan venom, as the intra-population variation exceeded the inter-population variation for enzymatic activity, procoagulant activity, and the abundance of neurotoxins. The pre-synaptic neurotoxin (taipoxin) was more abundant than the post-synaptic neurotoxins (3FTx), with a median of 11.0% (interquartile range (IQR): 9.7% to 18.3%; range: 6.7% to 23.6%) vs. a median of 3.4% (IQR: 0.4% to 6.7%; range: 0% to 8.1%). Three taipan individuals almost completely lacked post-synaptic neurotoxins, which was not associated with geography and occurred within two populations. We found no evidence of sexual dimorphism in taipan venom. Our study provides a basis for evaluating the significance of intra-specific venom variation within a phylogenetic context by comparing it to the inter-specific and inter-generic variation. The considerable intra-population variation we observed supports the use of several unpooled individuals from each population when making inter-specific comparisons.

## 1. Introduction

The coastal taipan *Oxyuranus scutellatus* is a medically important elapid. It has a mainly tropical distribution on the east coast of northern Australia (Queensland) and the south coast of New Guinea, with isolated populations in parts of north-western Australia, where there is higher precipitation [1,2]. They have an unusual ecology for an elapid, specializing in mammalian prey. Their diet consists mostly of rodents (72%), and then bandicoots (22%), with birds also being incorporated opportunistically (5%) [3]. The selective pressure caused by this ecological switch to a potentially dangerous mammalian prey source has resulted in a suite of morphological and prey handling specializations. These include a large body size (up to 2.9 m), large gape, long fangs, prodigious venom yield, highly potent venom, and a risk-minimizing “snap and release” biting strategy to reduce contact with their prey [3]. This hunting strategy requires a venom capable of extremely rapid immobilization to ensure that the prey can be efficiently located after release.

The composition of taipan venom is predominately phospholipase A2 (PLA2) toxins, three-finger toxins (3FTx), serine proteases (SVSP), kunitz peptides (KUN), metalloproteases (SVMP), cysteine-rich secretory proteins (CRiSP), and natriuretic peptides (NP) [4]. Bites to humans result in potentially fatal neurotoxicity and venom-induced consumption coagulopathy (VICC) [5]. Several important toxins have been purified from taipan venom and studied in detail. The presynaptic neurotoxin called taipoxin with a mass of approximately 45 kDa was first isolated in 1976 [6]. This is a trimeric PLA_2_ consisting of an α-subunit with a mass of 13.8 kDa, a β-subunit with a mass of 13.2 kDa, and a γ-subunit with a mass of 18.5 kDa [7]. Twenty years later, a post-synaptic neurotoxin, i.e., 6.8 kDa 3FTx, was isolated [8,9]. A procoagulant toxin, a serine protease called Oscutarin C, was isolated in 1986 and is a dimeric multi-domain prothrombin activator with a mass of approximately 300 kDa [10].

Recent advances in our understanding of snake phylogeny have made it possible to make phylogeny-based comparisons of snake venom proteomes. Linear parsimony can be used to determine if snake venom composition (or activity) co-varies with phylogenetic distance [11]. If closely related species with different diets have different venom proteomes, this would suggest positive selection for prey specific toxicity. However, if closely related species with similar diets had different venoms, then the explanation could be more complex. This may be the result of positive selection working on different toxins in the venom of each species, resulting in the evolution of divergent envenomation strategies, or it could be the result of genetic drift creating different venom phenotypes. Another variable could be that two species with similar diets have evolved different foraging strategies, which has led to highly divergent morphologies and in turn has resulted in fitness trade-offs, leading to different venom proteomes. It could also be a combination of any of the above.

In order to understand and interpret the significance of inter-specific venom variation in snakes, it is first necessary to investigate the intra-specific variability. Intra-specific venom variation can be classified into two types: inter-population variability—differences between populations; and intra-population variability—differences within populations (individual variation). Studies of snake venoms traditionally have used pooled venoms. Snakes of the same species, often from widely distant geographical regions and heterogeneous habitats, are milked and their venoms are then combined. This approach has resulted in a lack of data on the extent of intra-specific/intra-population venom variation in snakes, and may have contributed to an incomplete understanding of snake venom evolution. A small number of recent studies have examined inter- and intra-population venom variation, although these have been almost entirely restricted to New World crotalines [12,13,14,15,16]; or solely comparing the haemotoxic activity [17,18,19]. The results of these studies indicate that individual (intra-population) venom variation is genetically inherited and is not altered ontogenetically by environmental factors, seasonal variation, or diet [14,15,20,21,22]. An exception to this is ontogenetic venom changes in species with a bi-phasic juvenile to adult shift in dietary ecology [23,24,25]. Inter-population venom variation can be significant [13,26], and as venom is a trophic adaptation, these divergences are assumed to be the result of selection pressure due to local differences in diet, although this level of dietary data is usually lacking. Taipans are a dietary specialist, so all populations have a broadly similar diet, and it can reasonably be assumed that their venom composition would be fairly uniform throughout their distribution. This species is therefore potentially useful as a comparison for intra-specific venom studies on species with highly varied diets.

**Figure 1:**
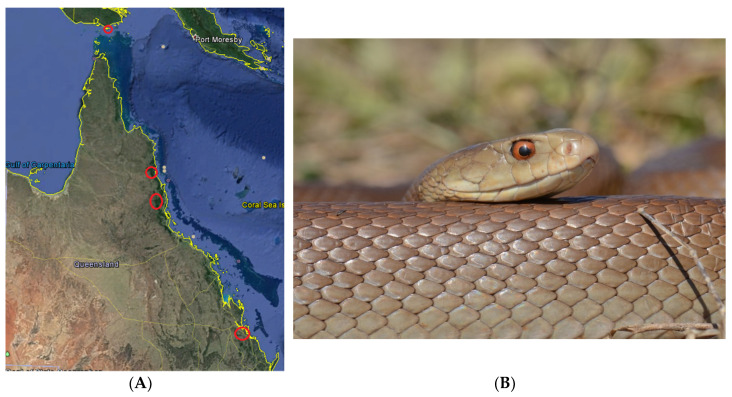
Panel (**A**), map of Queensland showing the localities of the taipans (*O. scutellatus*) sampled for our study. From south to north: Gladstone, Atherton Tableland, Cooktown, Saibai Island. Image taken from Google Earth. Panel (**B**), coastal taipan (*O. scutellatus*) from Atherton Tableland; photo courtesy Shane Black.

To investigate the intra-specific variation of coastal taipan venom, the venoms of 13 individual coastal taipans from four localities were compared—Gladstone, Atherton Tableland, Cooktown, and Saibai Island. These populations span a north-south distance of 2000 km (Figure 1). All the snakes used in the study were adults of known sex, and the venoms were compared both functionally and proteomically.

## 2. Results

### 2.1. Electrophoresis

**Figure 2:**
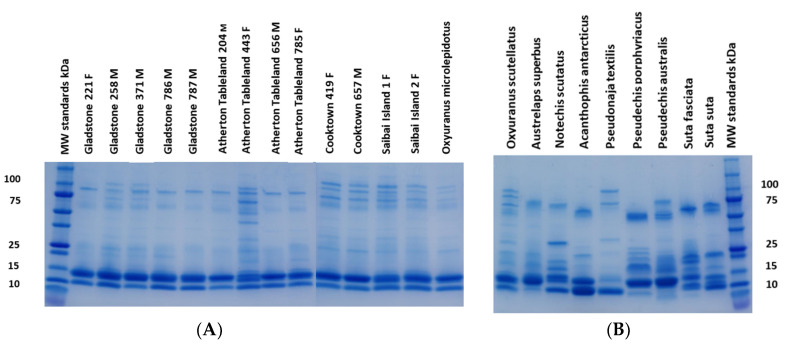
(**A**) One-dimensional sodium dodecyl sulfate-polyacrylamide gel electrophoresis (1D SDS-PAGE (reducing)) of the venoms from the 13 individual *O. scutellatus,* grouped into their respective populations. The lane on the far right is the congeneric species *O. microlepidotus.* M and F after the specimen I.D. codes at the top denote male or female. Numbers to the left of the gel are the molecular weights of protein standards in kDa. Note that the Mini-Protein TGX gels used have less resolution at low molecular weights, which is the likely reason why the 3FTxs with a molecular weight of 6.8 kDa are aggregated in the 10 kDa band. (**B**) 1D SDS-PAGE (reducing) of the pooled venoms from coastal taipan (far left lane) and eight other species of Australian elapids belonging to six genera: *Austrelaps superbus, Notechis scutatus, Acanthophis antarcticus, Pseudonaja textilis, Pseudechis australis, Suta fasciata*, and *S. suta* (numbers to the right of the gel are the molecular weights of the protein standards in kDa).

The variation observed in the electrophoretic venom profiles of the 13 coastal taipans (Figure 2a) was less than that observed in the eight other species of Australian elapids (Figure 2b). The electrophoretic profile of coastal taipans is highly distinctive and characterized by four main regions of staining. Their venom profiles are dominated by two intensely stained bands, a thin one at 10 kDa and a thick one at 14 kDa; then a series of four lightly stained bands spanning the region from approximately 60 to 110 kDa; and finally, one or two faintly stained bands in the region of 20 to 30 kDa.

One individual (C657) was chosen at random for a mass spectrometry analysis of the gel bands. This individual was used for the analysis of all the bands identified. The dominant band at 14 kDa was excised, trypsin digested, and its identity was confirmed by bottom-up mass spectrometry (MS) (orbitrap) as a basic PLA_2_, the alpha sub-unit of taipoxin. This toxin had a 52% sequence coverage and matched for 10 peptides, four of which were non-redundant. The next most intensely stained band (10 kDa) was shown by MS analysis to contain three toxins, including two peptides that matched to short neurotoxin 2 (3FTx) and a further two peptides matching to natriuretic peptide. Additionally, one peptide of high quality (95.3% confidence) matched a kunitz peptide (Figure S1). All the other bands on the gel were higher molecular weights—ranging from approximately 25 to 110 kDa. MS analysis of the bands at 25, 30, 37, 70, 90, and 100 kDa gave the highest confidence matches for all of these bands to the serine protease prothrombin activator Oscutarin C (9% to 22% sequence coverage and 5 to 65 peptides). The strongest match for this protein was the 30 kDa band (20% sequence coverage with 65 peptides matched). Other bands with faint levels of staining were present on the gel but were not investigated, so it is possible that other protein families are present in the venom of coastal taipan at much smaller amounts, not easily detectable by electrophoresis.

There did not appear to be any obvious visual differences between the males and females. All the individuals contained the characteristic four regions (10 kDa, 14 kDa, 20–30 kDa, and 60–110 kDa), with some variation present (e.g., 443F), and the Cooktown and Saibai Island individuals with more intensely stained bands in the 60–110 kDa region.

A comparison of eight other species of Australian elapids from six different genera showed a far greater range of variation in their venom profiles than the intra-specific variation observed in the individual taipans (Figure 2b). However, the venom profile of the congeneric species *O. microlepidotus* appeared to be within the range of variation of *O. scutellatus* (Figure 2a, far right lane).

### 2.2. Three-Finger Toxins (Post-Synaptic Neurotoxin)

**Figure 3:**
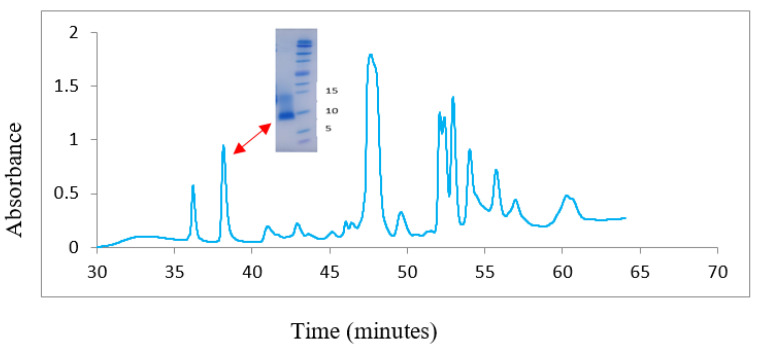
Reverse-phase high-performance liquid chromatography (RP-HPLC) chromatogram showing the location of the 3FTx peak (red arrow). Insert is the imaged 1D electrophoresis of this peak showing a single band suggesting purity that was confirmed with mass spectrometry (Figure S1). Flow rate was 0.2 mL/min, monitored at 215 nm.

**Figure 4:**
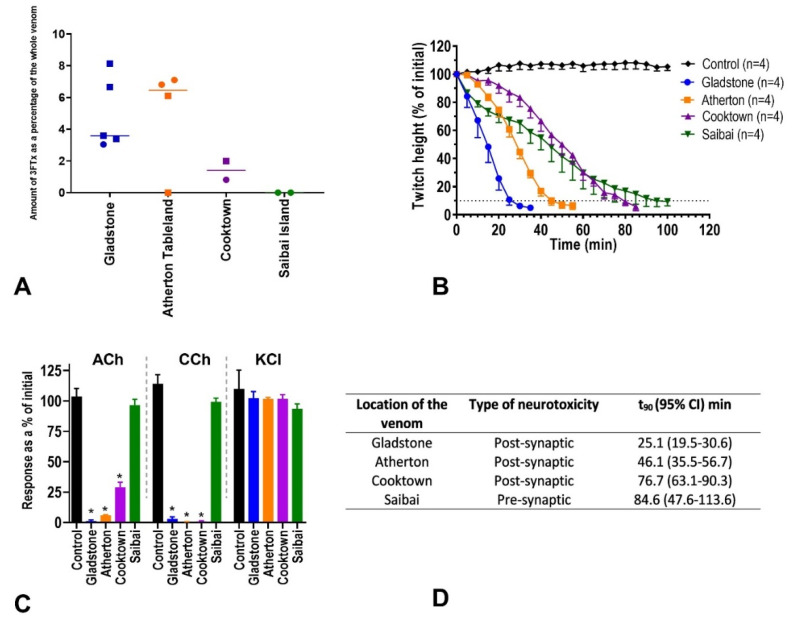
(**A**) The amount of post-synaptic neurotoxin (3FTx) present as a percentage of the whole venom (Y-axis) for all 13 *O. scutellatus*, as determined by the peak integration from reverse-phase high-performance liquid chromatography (RP-HPLC). The four populations are shown on the x-axis: Gladstone (blue), Atherton Tableland (orange), Cooktown (purple), and Saibai Island (green). Populations are subdivided by sex (squares = males, circles = females), with the median values included. (**B**) Inhibition of indirect twitches in isolated chick-biventer cervicis nerve-muscle preparation by taipan venoms (pooled populations) (5 µg/mL) for Gladstone (blue), Atherton Tableland (orange), Cooktown (purple), and Saibai Island (green) compared to the control (black). (**C**) The effect of pooled population taipan venoms on the response to exogenous agonists acetylcholine (ACh), carbachol (CCh), and KCI. Note: Saibai Island venom has a negligible effect on Ach and CCh, indicating presynaptic not postsynaptic neurotoxicity. (**D**) Middle column, nature of the neurotoxicity for each of the pooled populations, as determined by the contractile muscle response to the post venom application of nicotinic receptor agonists—i.e., pre- or post-synaptic. Right column, t_90_ values—time taken to cause a 90% reduction in twitch height (t_90_[min]; mean ± SD).

We quantified the amount of 3FTxs (post-synaptic neurotoxins) in the venoms of all individuals using reverse-phase high-performance liquid chromatography (RP-HPLC) (Figure 3). The location of this peak had previously been identified in a previous study [27]. The 3FTx peak commenced eluting between 38 and 41 min. It was the second peak to elute after the fluctuation in baseline following the injection spike (Figure 3, red arrow). The reason for the longer retention time in our study compared to the above-cited study was because we used a longer column. The purity and identity of this peak was confirmed for one individual chosen at random (G258) using SDS-PAGE/MS (Figure 3 insert and Figure S2). The intact mass spectrometry (LC-MS Q-TOF) of this toxin showed a molecular mass of 6.7 kDa (Figure S2). Based on the integration of the 3FTx peak, the median amount of 3FTx in the 13 venoms was 3.4% (interquartile range (IQR): 0.4% to 6.7%; range: 0 to 8.1%. Figure 4a). The 3FTx was absent or undetectable using RP-HPLC in three of the 13 individuals, and there was a high intra-population variation in the amount of 3FTx in each venom (Figure 4a). No sexual dimorphism was apparent in the amounts of this toxin (Figure 4a).

### 2.3. In-Vitro Neurotoxicity

Venoms of the individuals from each locality were pooled into four population venom pools for inter-population comparisons of neurotoxicity. All the venoms abolished indirect twitches within 100 min compared to the control, (Gladstone 35 min, Atherton 50 min, Cooktown 85 min, Saibai Island 100 min) (Figure 4b). All the pooled population venoms except for the venom from Saibai Island significantly inhibited the contractile responses to exogenous acetylcholine and carbachol compared to the control (one-way ANOVA, Dunnett’s multiple comparisons test, *p* < 0.05. Gladstone, Atherton, and Cooktown < 0.0001, Saibai Island 0.039) (Figure 4c). The t_90_means of the Gladstone, Atherton Tableland, Cooktown, and Saibai island venoms were 25.1, 46.1, 76.7, and 84.6 min, respectively (Figure 4d).

### 2.4. Taipoxin (Pre-Synaptic Neurotoxin)

**Figure 5:**
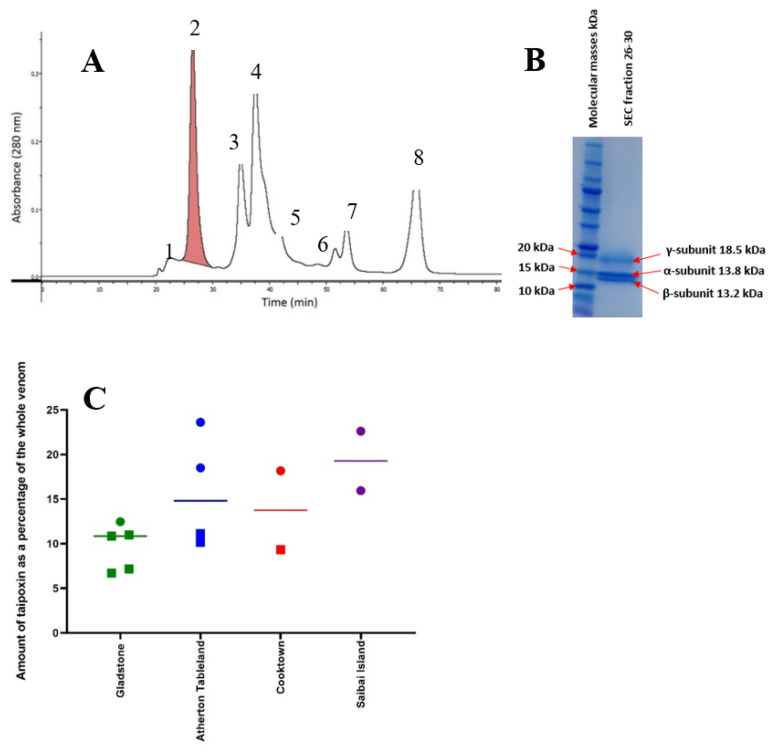
(**A**) Size-exclusion chromatogram of *O. scutellatus* (Atherton Tableland 785F), with the venom monitored at 280nm and a flow rate of 0.4mL/min. The peak identified as taipoxin is the first large peak eluting between 26 and 30 min (shaded red—peak 2). (**B**) 1D SDS-PAGE reducing gel of the red-shaded peak 2. The three subunits of taipoxin are clearly visible with no other staining apparent, indicating a pure fraction. (**C**) Scatter plot (with medians) of the amounts of taipoxin for each of the 13 taipans as a percentage of the whole venom (y-axis), as determined by size-exclusion chromatography. X-axis is the individual snakes grouped and color-coded into their respective populations: Gladstone (green), Atherton Tableland (blue), Cooktown (red), and Saibai Island (purple), with males (squares) and females (circles).

To quantify the amount of taipoxin present, the venom of all the individuals was subjected to Size-Exclusion Chromatography (SEC). The fractionation of *O. scutellatus* venom by size-exclusion chromatography resulted in eight peaks (Figure 5a). The first large peak (peak 2) with an elution time of 26 to 30 min has been previously identified as the pre-synaptic PLA_2_ neurotoxin taipoxin [6,7,27,28]. Based on the integration of the peak, the median amount of taipoxin in the venoms was 11.0% (IQR: 9.7% to 18.3%; range: 6.7% to 23.6%. Figure 5c). The purity of the peak was confirmed with a 1D SDS-PAGE, which showed the three sub-units and no other staining (Figure 5b). There was no geographic variation in the abundance of this toxin; a Kruskal–Wallis test analysis showed that the population medians did not vary significantly (*p* value 0.2068) (Figure 5c).

### 2.5. Coagulation Assay

#### 2.5.1. Human Plasma

**Figure 6:**
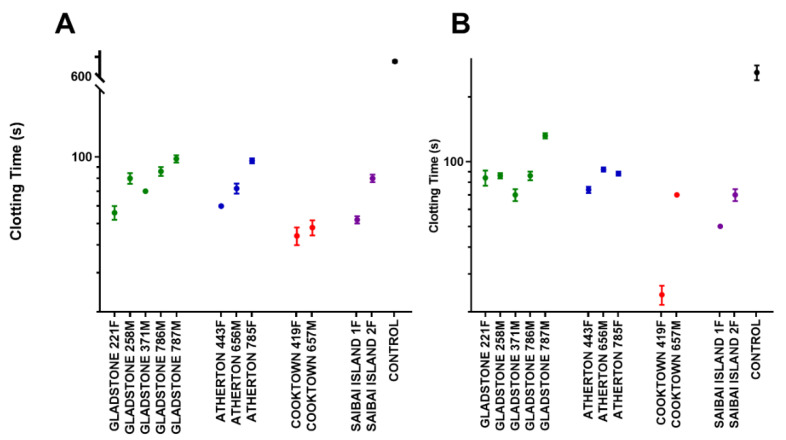
(**A**) Mean (standard error) of the clotting times for each individual snake venom (50 ng/mL; *n* = 5 assays) in human plasma, grouped by population: Gladstone (green), Atherton Tableland (blue), Cooktown (red), Saibai Island (purple), and control (black; *n* = 8). Clotting time was monitored at 10 s intervals. (**B**) Mean (standard error) of the clotting times for each individual snake venom (500 ng/mL; *n* = 5 assays) in rat plasma, grouped by population: Gladstone (green), Atherton Tableland (blue), Cooktown (red), Saibai Island (purple), and control (black; *n* = 6). Clotting time was monitored at 10 s intervals.

The venom (50 ng/mL) from all 13 individual snakes decreased the clotting time in human plasma, with a median clotting time of 70 s (range: 50 to 100 s), and was significantly different to the clotting time of 720 s when the venom was not present (*p* < 0.0001; Kruskal–Wallis test; Figure 6a). There was no significant inter-population variability in the clotting times or difference between sexes (Figure 6a).

#### 2.5.2. Rat Plasma

A venom concentration 10 times that of human plasma was used in rat plasma, based on a previous study [29]. Venom (500 ng/mL) from all 13 individual snakes decreased the clotting time in rat plasma, with a median clotting time of 76 s (range 20 to 140 s) compared to a clotting time of 255 s when venom was not present (*p* < 0.0001; Kruskal–Wallis test; Figure 6b). No association was evident between the rat plasma procoagulant activity and either sex or population/geography.

### 2.6. PLA_2_ Assay

**Figure 7:**
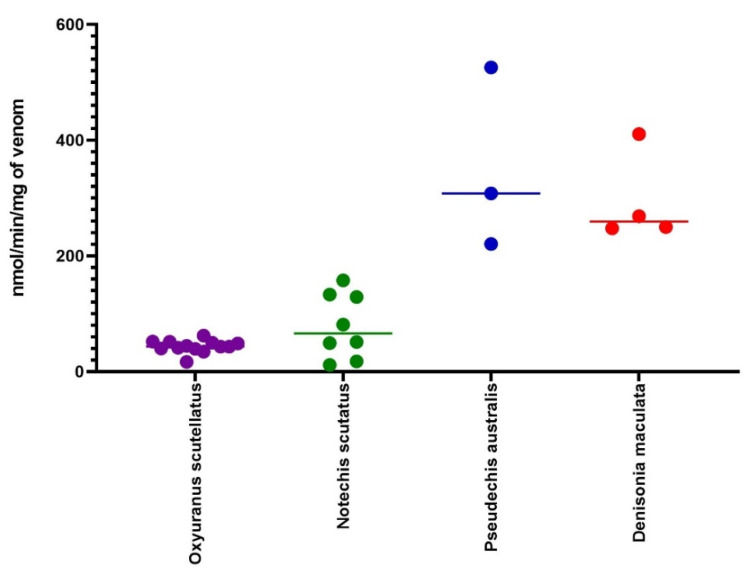
A scatter plot of PLA_2_ activity in taipans (*O. scutellatus*; *n* = 13; purple), tiger snake (*N. scutatus*; *n* = 8;green), mulga snake (*P. australis*; *n* = 3; blue), and ornamental snake (*D. maculata*; *n* = 4; red). All the assays were performed *n* = 5 and then averaged.

PLA_2_ assays were performed on the venoms of all individuals. The median PLA_2_ activity was 43 nanomoles of chromophore produced per minute per mg of venom (nmol/min/mg) (range: 17 to 62 nmol/min/mg). There was minimal variation in the PLA_2_ activity between the individuals and populations of taipans compared to three other genera of Australasian elapids (Figure 7).

### 2.7. L-Amino Acid Oxidase Assay

There was a complete absence of L-amino acid oxidase (LAAO) activity in the venoms from all 13 individual snakes (Figure S3).

## 3. Discussion

We found that the intra-specific variation in taipan venom was less than the inter-specific variation between taipan and eight other species of Australian elapids (Figure 2 and Figure 7). Additionally, the intra-population variation in taipans was greater than the inter-population variation. This was demonstrated for the SDS-PAGE, quantified amounts of taipoxin and 3FTx, clotting activity, and PLA_2_ activity. An electrophoretic analysis of taipan venom showed that it has a characteristic venom profile, which is easily distinguishable from other genera of Australian elapids, but not from its congener *O. microlepidotus,* Inland Taipan (Figure 2).

Our study provides a basis for evaluating the degree and significance of intra-specific variation within a phylogenetic context by comparing it to the variability within genera and between genera of snakes. Variability can arise from many sources, and it is important to disentangle random variability from identifiable sources. Some important factors to consider when planning studies on intra-specific variation are; the degree of genetic isolation between populations, the degree of geographical variation in diets, and the venom potency of the species in question, as weaker venom would presumably be under a stronger selection pressure. We chose the highly venomous dietary specialist coastal taipan for our study, because of the similar diet between all the populations and the minimal genetic isolation for population divergence to occur. This should have eliminated many of the identifiable variables. Low intra-population and higher inter-population variation in gene frequencies is characteristic of genetic drift in conspecific populations [30]. As we observed a high intra-population and lower inter-population variation, genetic drift was not responsible for the variation in this study, which is presumably the result of random genetic variation within populations. Previous studies on intra-specific snake venom variation have focused on sexual dimorphism [20,21,31,32], ontogenetic changes [13,15,21,32,33], and whether venom composition can be altered by the captive conditions [20,22,34]

A few previous studies have investigated the degree of variation between populations both pooled [34,35,36], or compared intra-population variation with inter-population variation [15,37,38,39]. Two previous studies have presented results combining intra-specific variation with inter-specific variation [12,40]. One of these [40] compared three pooled populations of *Naja kaouthia* with 10 other species of cobras—so it was a within-clade comparison. The amount of intra-specific variation in this species was less than the amount of inter-specific variation between the 10 species of cobras, similar to our study of taipans.

Another study comparing four subspecies each of two species of rattlesnakes (*Crotalus lepidus* and *C. willardi* [12] found remarkably conserved electrophoretic profiles for the four subspecies of *C. willardi*. In contrast, for *C. lepidus* the two northern subspecies (*C.l.klauberi* and *C.l.lepidus*) were readily distinguishable from the two southern subspecies (*C.l maculosus* and *C.l. morulus*), but very similar to one another. The two southern populations were also similar but distinguishable from each other by the amount of PLA_2_ staining. The range of intra-specific variation in *C. lepidus* may come close to exceeding the degree of inter-specific variation between it and *C. willardi.* There are complex evolutionary and ecological reasons for why this may occur in some snake species. Similarly, we could not distinguish coastal taipan from the inland taipan (*O. microlepidotus*), one of its congeners, electrophoretically.

Our results suggest that the extent of intra-specific venom variation is negligible in the context of inter-generic comparisons, but in some instances it may be a consideration when making within-genus comparisons.

The high level of intra-population variation we observed indicates that making allowances for this aspect of snake venom may be advisable when planning inter-specific venom variation comparisons. Pooling populations is problematic, as if different individual snakes express different toxins it will create a falsely complex picture of the venom complexity. Perhaps using several unpooled individuals from each population would be the optimal methodology.

The results of our clotting assays showing minimal inter-population variation in taipan venom corroborate and expand upon a previous study [41], which used single individuals from three of the same populations.

There was a virtual absence of post-synaptic neurotoxins (3FTxs) in three of the taipans, as determined by the RP-HPLC. This absence was not associated with geography or sex, but appeared to be the result of expression variation within the populations. This absence of 3FTx was corroborated with in-vitro neurotoxic assays using the chick biventer cervicis nerve muscle preparation. After the application of exogenous acetycholine to the venom from the Saibai Island population (which lacked 3FTx), the nictotinic acetycholine receptors were almost fully functional, indicating that they were not inhibited (Figure 4c)—post-synaptic blockade. In contrast, the venom from populations with relatively higher levels of 3FTx (Gladstone and Atherton Tableland) resulted in almost complete nicotinic acetylcholine receptor blockade. This high individual variability in the abundance of post-synaptic neurotoxins contrasted with the relatively similar amounts of pre-synaptic neurotoxins in individual snake venoms. The finding of such a high percentage of individuals lacking 3FTx expression suggests a degree of redundancy in taipan venom. Perhaps the presence of taipoxin and Oscutarin C alone confers a sufficient fitness benefit without the need for the fast-acting post-synaptic neurotoxin. Further studies examining this aspect of taipan venom may be illuminating, as would elucidating the exact mechanism causing the lack of 3FTx expression. We found no evidence of sexual dimorphism in the venom of *O. scutellatus*.

### Limitations and Further Research

The limitations of our study were the small sample size from each population and the inability to sample specimens from all parts of the taipan’s distribution—e.g., extreme south-east QLD, Kimberley, Top End, and New Guinea. Future research into the mechanism causing the lack of 3FTx expression in some individuals will be important. On a larger scale, future study designs incorporating two highly venomous species, one a dietary generalist and one a dietary specialist; and two mildly venomous species, one a dietary generalist and one a dietary specialist, would test some of the ideas expressed in this study. Finally, it should be emphasized that none of the localities sampled for this study represent true genetically isolated populations. All would be influenced by gene migration, so they are technically meta-populations [42].

## 4. Materials and Methods

### 4.1. Materials

Thirteen snakes were used in the study: Gladstone: OS786 male, OS787 male, OS 371 male, OS258 male, OS 221 female. Atherton Tableland: OS443 female, OS204 male, OS656 male, OS785 female. Cooktown; OS419 female, OS657 male. Saibai Island: OSC1 female, OSC2 female. All the venoms were obtained from a single milking of an individual snake. Milkings were carried out on two separate days, in November 2016 at Venom Supplies South Australia. Each milking was kept separate and given a reference I.D. number and stored at −80 °C, until all the snakes were milked and then all the venoms were freeze-dried. All the experiments were conducted with individual venoms, except the in-vitro neurotoxic assays, for which the venoms were pooled into populations to minimize the number of animals killed. Small quantities (approx. 1 mg) of lyophilized venom were weighed out and reconstituted on the day of the experiments to ensure there was no loss of enzyme activity due to degradation in solution.

Mini-PROTEAN Tetra Cell, 2-Gel System (BIO-RAD # 1658005). Mini-PROTEAN TGX Gels (BIO-RAD # 456-9034). Precision Plus Protein dual color standard. Criterion cell #1656001 Criterion precast gels 16.5% Tris-Tricine #3450064. Tricine sample buffer #161-0739. Grass S88 stimulator (Grass Technologies, West Warwick, RI, USA). Acetylcholine (Sigma-Aldrich, St Louis, MO, USA), carbochol (Sigma). Column; Phenomenex Jupiter C18 (250 mm × 4.6 mm), bead size 5µm, 300Å. Acetonitrile 190 grade Item#20060.320, Trifluoroacetic acid. Q Exactive Plus Hybrid Quadrupole-Orbitrap Mass Spectrometer (Thermo Fischer Scientific, Waltham, MA, USA) and a SCIEX 6600 TripleToF (QToF) (SCIEX, Sydney, Australia). Column; Superdex 75 10/300GL. Ammonium Acetate A1542-250G Sigma Life Sciences, Australian Red Cross Fresh Frozen Plasma #596014, CaCl_2_, rat plasma. Tris (Hydromethyl methylamine) 2311-500G Ajax Finechem. NaCl AJA465-500G Ajax Finechem, 4-nitro-3-octanoyloxybenzoic acid (NOB) Cat. No. BML-ST506-0050 Enzo Life Sciences. Leucine Pcode 1001836926 L800-25G Sigma Life Sciences, Ortho-dianisidine Pcode 1001844919, Horseradish peroxidase Pcode 1002325511. Christ 3-4 alpha-LSC basic freeze-dryer.

### 4.2. Sodium Dodecyl Sulfate Polyacrylamide Gel Electrophoresis (SDS-PAGE)

An amount of 16 µL of a (3:1 ratio) venom/sample buffer solution was loaded into each well. The venom concentration was 1 mg/mL, so there was 12 µg of venom per well. Reducing conditions were 95 °C for 4 min. Gels were run at 100 V at room temperature and stopped when the dye front was less than 10 mm from the base of the gel. Gels were stained with 0.1% (*w*/*v*) Coomassie brilliant blue and destained in millipure water. The gels were then imaged with an Amersham Imager 600 GE. Electrophoresis was performed with both 4–20% Mini-Protean TGX gels and Criterion Tris/tricine gels.

### 4.3. Reverse-Phase High Performance Liquid Chromatography (RP-HPLC)

The column used was a Phenomenex Jupiter C18 column (250 × 4.6 mm, 5 µm, 300 Å). The pump used was a Shimadzu LC-20AD, and the elution of peaks was monitored with a Shimadzu SPD-20A detector with absorbance monitored at 214 nm. Mobile phase A—water with 0.1% trifluoroacetic acid (TFA); mobile phase B—90% acetonitrile with 0.09% TFA, flow rate 0.2 mL/min; gradient 0% to 20% over 5 min, 20% to 60% between 5 min and 40 min, then 60% to 80% between 40 min and 45 min, and finally 80% to 0% between 45 min and 50 min. Lyophilized venom was reconstituted in Millipure water at a concentration of 1mg/mL. The injection volumes were 200 µL. Before applying venom, the column was first equilibrated with 5% mobile phase B. Software processing and analysis was performed using LabSolutions (2010–2017 Shimadzu Corporation). GraphPad Prism version 8.3.1 software (GraphPad software Inc., La Jolla, CA, USA) was used for statistical analyses and data presentation. For all statistical tests, *p* < 0.05 was considered statistically significant. The Kruskal–Wallis test was used for comparing populations. Comparisons were expressed as medians and inter-quartile ranges.

### 4.4. Size-Exclusion Chromatography (SEC)

SEC was used to isolate the multimeric toxin taipoxin, as RP-HPLC would have caused this toxin to break apart. The column used was a Superdex 75 10/300GL. The pump, detector, and software were as above as for RP-HPLC (Section 4.3), except that absorbance was monitored at 280 nm. The column was equilibrated with distilled water with a flow rate of 0.4 mL/min for 15 min, then for 60 min with a buffer consisting of 0.05 M NaCl (5 mL) + 0.1 M ammonium acetate (20 mL) + H_2_0 (75 mL). The column was then further equilibrated with a buffer consisting of 0.15 M NaCl (37.5 mL) + 0.1 M ammonium acetate (50 mL) and H_2_0 (162.5 mL) for 74 min at a flow rate of 0.65 mL/min. The venom sample was then spiked into this buffer at a reduced flow rate of 0.4 mL/min. The venom concentration was 5 mg/mL; 200 µL of venom sample was spiked to give an amount of 1 mg of venom in the column. Software processing and analysis was performed using LabSolutions (2010–2017 Shimadzu Corporation). The GraphPad Prism version 8.3.1 software (GraphPad software Inc., La Jolla, CA, USA) was used for the statistical analyses and data presentation. For all the statistical tests, *p* <0.05 was considered statistically significant. The Kruskal–Wallis test was used for comparing the populations and the Mann Whitney test was used for comparing sexes. The comparisons were expressed as medians and inter-quartile ranges.

### 4.5. Isolated Chick Biventer Cervicis Nerve-Muscle Preparation

Chicks aged 4 to 10 days were euthanized with CO_2_. After dissection, the biventer cervicis nerve-muscle preparations were mounted under 1 g tension in 5 mL organ baths containing physiological salt solution (NaCl, 118.4 mM; KCl, 4.5 mM; MgSO_4_, 1.2 mM; KH_2_PO_4_, 1.2 mM; CaCl_2_, 2.5 mM; NaHCO_3_, 25 mM; and glucose, 11.1 mM). The organ baths were maintained at 34 °C and bubbled with carbogen (95% O_2_; 5% CO_2_). Electrical stimulation (0.2 ms duration, 0.1 Hz, supramaximal V) evoked indirect twitches. The stimulation was ceased, and the responses to acetylcholine (ACh, 1mM for 30 s), carbochol (CCh, 20 µM for 60 s), and potassium chloride (KCl, 40 mM for 30 s) were obtained. The organ bath was then thoroughly washed, and electrical stimulation was recommenced and maintained for 30 min to allow the preparation to equilibrate. Venom (5 µg/mL) was added to the organ bath and the twitch height was recorded until the abolition of twitch response, or stopped after 3 h. A further application of ACh, CCh, and KCl (as above) was then performed to obtain a contractile response. The twitch responses to electrical stimulations and contractile responses to agonists (ACh, CCh, and KCI) were measured using a Grass FT03 force displacement transducer (Grass Instruments, Quincey, MA, USA) and recorded on a PowerLab system (ADInstruments Pty Ltd., Bella Vista, NSW, Australia). The time taken for the 90% inhibition of the maximum twitch response to occur (t_90_ values) was determined for each of the venom samples. The twitch and contractile responses were expressed as percentages of their pre-venom values. A one-way ANOVA followed by a Tukey’s multiple comparison post-test were used to compare the responses to exogenous agonists following the administration of venom. Data are presented as mean ± standard error of the mean (S.E.M) of four experiments. GraphPad Prism version 8.3.1 software (GraphPad software Inc., La Jolla, CA, USA) was used for the statistical analyses and data presentation. For all statistical tests, *p* < 0.05 was considered statistically significant.

### 4.6. Intact Mass Spectrometry

NanoLC–MS/MS was performed using a Dionex UltiMate 3000RSLC nanoflow HPLC system (Thermo Fisher Scientific). Thelyophilized venom fractions eluting from the capillary LC were resuspended in buffer A (0.1% formic acid) and directly loaded onto an Acclaim PepMap100 C18 75 μm × 20 mm trap column (Thermo Fisher Scientific) for pre-concentration and online desalting. Separation was then achieved using an EASY-Spray PepMap C18 75 μm× 500 mm column (Thermo Fisher Scientific), employing a linear gradient from 2 to 32% acetonitrile at 300 nL/min over 120 min. Q-Exactive Plus MS System (Thermo Fisher Scientific) was operated in full MS/data dependent acquisition MS/MS mode. The Orbitrap mass analyzer was used at a resolution of 120,000 to acquire a full MS with an *m*/*z* range of 390–1400, incorporating a target automatic gain control value of 1 × 10^6^ and a maximum fill time of 50 ms. The acquired data were converted to peak lists using Xcalibur (Thermo Fisher Scientific). By using the mass tolerances in MS of 10 ppm, the peaks were deconvoluted, and the intact mass was manually calculated using the mass and the charge state of the protein.

### 4.7. Mass Spectrometry (LC-MS/MS)

The fractionation of the material was achieved by dissecting uniform pieces of the gel (1 mm wide bands). In total, this achieved 9 bands from each fraction. Individual polyacrylamide gel plugs were washed 5 times with 25 mM ammonium bicarbonate in 50% methanol. The gel slices were shrunk by leaving them overnight at room temperature. The plugs were reconstituted with 50 mM of ammonium bicarbonate containing 800 ng of trypsin (Promega Corporation, Madison, WI, USA) and left at room temperature for 5 h. The resulting peptides were extracted by the double application of 20 mL of 50% ACN /0.1 % trifluroacetic acid, and the extracts were combined.

MS analysis was performed using the nanoLC-Ultra^TM^(Eksigent) system operating with both a trapping and resolving column. In each run, 1 mL of the tryptic digested peptides was loaded onto the trap column (200 mm × 0.5 mm Chrom XP C18-CL, 3 mM, 120 Å), and washed for 5 min at 2 mL/min with 2% mobile phase B (Mobile phase A consisting of 0.1% formic acid and mobile phase B consisting of 0.1% formic acid and 80% ACN). The sample was then eluted directly on the reversed phase column (75 mm × 15 cm ChromXP, C18, 3 mm, 120 Å) using a linear gradient from 7% B to 30% B at a running speed of 300 nL/min over 90 min. The eluted peptides were analyzed in either the data-dependent or SWATH acquisition mode in a Time-of-Flight mass spectrometer (TripleToF 6600 system, SCIEX, Sydney, Australia).

The spectral library was constructed using 6 separate runs from the data-dependent mode. Herein, 20 of the most intense peaks found in the MS survey scan covering *m*/*z* 400–1250 were selected for MS/MS fragmentation. The precursors were then excluded for 30 s after being selected and *m*/*z*-dependent collision energy setting with the energy spread of 5 eV was used. The data-dependent files were searched through the ProteinPilot^TM^ v 5.0 software (SCIEX, Singapore), with SwissProt database to generate the peptide spectral library for identification. using a False Detection Rate (FDR) threshold of 1%.

### 4.8. Procoagulant Assay

The procoagulant activity was determined by a turbidimetric assay developed by O’Leary and Isbister [43]. The venom was reconstituted in water at a concentration of 50 ng/mL. This was the weakest concentration that gave reliable results. One row of wells in a 96-well microplate each had 100 µL of venom (5ng per well) placed in them. An amount of 1 mL of plasma (Australian Red Cross fresh frozen plasma #2575195) was combined with 0.4M of CaCl_2_ (20 µL/mL), and then 100 µL of this plasma and calcium was then added to all the wells simultaneously with a multi-channel pipette. The reaction was monitored in a spectrophotometer at 340 nm at 37 °C. Readings were taken every 10 s. A negative control well contained 100 µL of water (and plasma). Rat plasma clotting assays were performed the same way, except that the venom concentration was 500 ng/mL (due to the higher resistance of rat plasma to snake venom procoagulant toxins), and each well only contained 50 µL of both venom and plasma. For the rat plasma assays, 10 µL/mL of CaCl_2_ was used. GraphPad Prism version 8.3.1 software (GraphPad software Inc., La Jolla, CA, USA) was used for the statistical analyses and data presentation. For all the statistical tests, *p* <0.05 was considered statistically significant. The Kruskal–Wallis test was used for comparing populations.

### 4.9. PLA_2_ Assay

The Phospholipase A_2_ (PLA_2_), activity was determined using a synthetic chromogenic substrate 4-nitro-3-octanoyloxybenzoic acid (NOB). An amount of 10 mL of NOB buffer (10 mM Tris, 100 mM NaCl, 10 mM CaCl_2_, pH 8), was made up in water. The microplate wells were prepared with 100 µL of venom (1 mg/mL, reconstituted in NOB buffer), then 100 µL of NOB buffer containing 194 nmol NOB substrate was added. The venom and substrate were preheated at 37 °C for 10 min, then mixed and the reaction monitored on a plate reader at 425 nm. A standard curve was created by the alkaline hydrolysis of NOB substrate using 4 M of NaOH, and the activity was measured as the nanomoles of product released per minute per mg venom. GraphPad Prism version 8.3.1 software (GraphPad software Inc., La Jolla, CA, USA) was used for the statistical analyses and data presentation. For all statistical tests, *p* < 0.05 was considered statistically significant. The Kruskal–Wallis test was used for comparing populations.

### 4.10. L-amino Acid Oxidase Assay

To determine the L-amino acid oxidase activity, 50 µL of venom (1 mg/mL) was added to 100 µL of reagent mixture (50 µL Leucine (10 mg/mL), 100 µL O-dianisidine (1 mg/mL), 10 µL Horseradish peroxidase (0.75 mg/mL), 2 mL 25 mM Tris pH 8.5), and the reaction was monitored at 450 nm. Venom from a snake species known to possess L-amino acid oxidase activity (*Suta suta* 100 µg/mL) was used as a positive control, and Tris buffer was used as a negative control.

## Supplementary Materials

The following are available online at https://www.mdpi.com/2072-6651/12/8/485/s1: Figure S1: Mass spectrometry results of the trypsin digest of the 10 kDa peak showing the presence of kunitz peptides and natriuretic peptides in addition to short-chain neurotoxins. Figure S2: Intact mass spectra of 38 min peak on Q-Exactive Orbitrap showing the mass of the monoisotopic peak to be 6.7 kDa corresponding to the mass from 3FTxs previously recorded from *O. scutellatus*. Figure S3: LAAO assay of all 13 taipans with venom of the elapid *Suta suta* as a positive control (red).
