# Supplementary material for: Intra-Specific Venom Variation in the Australian Coastal Taipan Oxyuranus scutellatus
Source: Toxins (Basel). 2020 Jul 30;12(8):485. doi: 10.3390/toxins12080485 (PMC7472000; doi:10.3390/toxins12080485)
Supplement: Supplementary file 1 [file toxins-12-00485-s001.pdf]

Supplementary Materials: Intra-Specific Venom Variation in the Australian Coastal Taipan *Oxyuranus scutellatus*

Theo Tasoulis, Anjana Silva, Punnam Chander Veerati, Mark Baker, Wayne C. Hodgson, Nathan Dunstan and Geoffrey K. Isbister

Electrophoresis/MS

<table>
<tr><th>B</th><th>C</th><th>D</th><th>E</th><th>F</th><th>G</th><th>H</th><th>I</th><th></th></tr>
<tr><td>Best N (Pe</td><td>All N</td><td>Accessions</td><td>Names</td><td>Start Pc</td><td>Best Conf (Pe</td><td>Best Hypo</td><td>Sequence</td><td>Mo</td></tr>
<tr><td>1</td><td>1</td><td>sp|P0CB06|3S12_OXYSC; sp|Q45Z11|3S11_O</td><td>Short neurotoxin 2 OS=Oxyuranus scutellatus</td><td>1</td><td>97</td><td>96.97</td><td>MTCYNQQSSEAK</td><td>Met</td></tr>
<tr><td>1</td><td>1</td><td>sp|P0CB06|3S12_OXYSC; sp|Q45Z11|3S11_O</td><td>Short neurotoxin 2 OS=Oxyuranus scutellatus</td><td>1</td><td>32.3</td><td>32.32</td><td>MTCYNQQSSEAK</td><td>Oxi</td></tr>
<tr><td>1</td><td>1</td><td>sp|P0CB06|3S12_OXYSC; sp|Q45Z11|3S11_O</td><td>Short neurotoxin 2 OS=Oxyuranus scutellatus</td><td>1</td><td>32.3</td><td>32.02</td><td>MTCYNQQSSEAK</td><td>Oxi</td></tr>
<tr><td>1</td><td>1</td><td>sp|P0CB06|3S12_OXYSC; sp|Q45Z11|3S11_O</td><td>Short neurotoxin 2 OS=Oxyuranus scutellatus</td><td>1</td><td>32.3</td><td>28.05</td><td>MTCYNQQSSEAK</td><td>Carl</td></tr>
<tr><td>1</td><td>1</td><td>sp|P0CB06|3S12_OXYSC; sp|Q45Z11|3S11_O</td><td>Short neurotoxin 2 OS=Oxyuranus scutellatus</td><td>1</td><td>32.3</td><td>16.09</td><td>MTCYNQQSSEAK</td><td>Carl</td></tr>
<tr><td>1</td><td>1</td><td>sp|P0CB06|3S12_OXYSC; sp|Q45Z11|3S11_O</td><td>Short neurotoxin 2 OS=Oxyuranus scutellatus</td><td>1</td><td>32.3</td><td>15.94</td><td>MTCYNQQSSEAK</td><td>Carl</td></tr>
<tr><td>1</td><td>1</td><td>sp|P0CB06|3S12_OXYSC; sp|Q45Z11|3S11_O</td><td>Short neurotoxin 2 OS=Oxyuranus scutellatus</td><td>1</td><td>99</td><td>90.45</td><td>MTCYNQQSSEAK</td><td>Carl</td></tr>
<tr><td>1</td><td>1</td><td>sp|P0CB06|3S12_OXYSC; sp|Q45Z11|3S11_O</td><td>Short neurotoxin 2 OS=Oxyuranus scutellatus</td><td>1</td><td>99</td><td>2.234</td><td>MTCYNQQSSEAK</td><td>Carl</td></tr>
<tr><td>1</td><td>1</td><td>sp|P0CB06|3S12_OXYSC; sp|Q45Z11|3S11_O</td><td>Short neurotoxin 2 OS=Oxyuranus scutellatus</td><td>1</td><td>92.5</td><td>92.45</td><td>MTCYNQQSSEAK</td><td>Carl</td></tr>
<tr><td>1</td><td>1</td><td>sp|P0CB06|3S12_OXYSC; sp|Q45Z11|3S11_O</td><td>Short neurotoxin 2 OS=Oxyuranus scutellatus</td><td>1</td><td>80.3</td><td>80.31</td><td>MTCYNQQSSEAK</td><td>Met</td></tr>
<tr><td>1</td><td>1</td><td>sp|P0CB06|3S12_OXYSC; sp|Q45Z11|3S11_O</td><td>Short neurotoxin 2 OS=Oxyuranus scutellatus</td><td>1</td><td>80.3</td><td>71.95</td><td>MTCYNQQSSEAK</td><td>Thr-</td></tr>
<tr><td>1</td><td>1</td><td>sp|P0CB06|3S12_OXYSC; sp|Q45Z11|3S11_O</td><td>Short neurotoxin 2 OS=Oxyuranus scutellatus</td><td>3</td><td>99</td><td>99</td><td>CYNQQSSEAK</td><td>Cys-</td></tr>
<tr><td>1</td><td>1</td><td>sp|P0CB06|3S12_OXYSC; sp|Q45Z11|3S11_O</td><td>Short neurotoxin 2 OS=Oxyuranus scutellatus</td><td>3</td><td>99</td><td>86.5</td><td>CYNQQSSEAK</td><td>Cys-</td></tr>
<tr><td>2</td><td>2</td><td>sp|B7S4N9|VKT_OXYSC</td><td>Kunitz-type serine protease inhibitor taicotox</td><td>47</td><td>95.3</td><td>95.3</td><td>FYYNPHSK</td><td></td></tr>
<tr><td>3</td><td>3</td><td>sp|Q3SAX8|VNPD_OXYSC</td><td>Natriuretic peptide OsNP-d (Fragment) OS=O:</td><td>22</td><td>38.3</td><td>38.32</td><td>IGSVSGLGCNR</td><td>Carl</td></tr>
<tr><td>3</td><td>3</td><td>sp|Q3SAX8|VNPD_OXYSC</td><td>Natriuretic peptide OsNP-d (Fragment) OS=O:</td><td>22</td><td>47.3</td><td>47.29</td><td>IGSVSGLGCNR</td><td>Carl</td></tr>
<tr><td>3</td><td>3</td><td>sp|Q3SAX8|VNPD_OXYSC</td><td>Natriuretic peptide OsNP-d (Fragment) OS=O:</td><td>33</td><td>55.4</td><td>55.42</td><td>LVQNPPKPISGES</td><td>Asn</td></tr>
<tr><td>3</td><td>3</td><td>sp|Q3SAX8|VNPD_OXYSC</td><td>Natriuretic peptide OsNP-d (Fragment) OS=O:</td><td>33</td><td>93.7</td><td>93.66</td><td>LVQNPPKPISGES</td><td></td></tr>
<tr><td>3</td><td>3</td><td>sp|Q3SAX8|VNPD_OXYSC</td><td>Natriuretic peptide OsNP-d (Fragment) OS=O:</td><td>33</td><td>93.7</td><td>55.09</td><td>LVQNPPKPISGE</td><td>Oxi</td></tr>
<tr><td>3</td><td>3</td><td>sp|Q3SAX8|VNPD_OXYSC</td><td>Natriuretic peptide OsNP-d (Fragment) OS=O:</td><td>33</td><td>93.7</td><td>41.77</td><td>LVQNPPKPISGES</td><td>Oxi</td></tr>
<tr><td></td><td></td><td></td><td></td><td></td><td>99</td><td>99</td><td>AEESSGPAAGR</td><td></td></tr>
<tr><td></td><td></td><td></td><td></td><td></td><td>37.4</td><td>37.41</td><td>NWNINTK</td><td></td></tr>
</table>

Figure S1. Mass spectrometry results of the trypsin digest of the 10kDa peak showing the presence of kunitz peptides and natriuretic peptides in addition to short-chain neurotoxins.

Mass Spectrometry

Mass spectrometry (SCIEX 6600 TripleTof) of this band gave a 78% sequence coverage at 95% confidence as being a 3FTx neurotoxin previously recorded in *O. scutellatus* venom.

Fraction 38 min from the RP-HPLC was injected directly into the Q-Exactive Orbitrap. The mass spectra and intact mass spec were recorded for at least 5 min. The spectra were then averaged, and the monisotopic peak was found to be 1345.2 Da (z = 5) (Figure S2. The protein is 6.7 kDa.

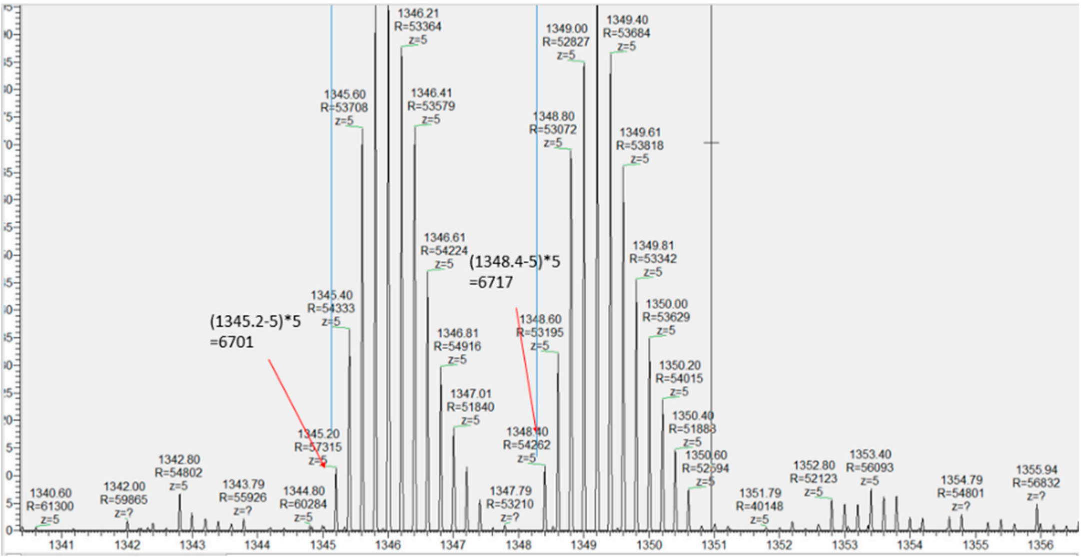

Figure S2. Intact mass spectra of 38 min peak on Q-Exactive Orbitrap showing the mass of the monoisotopic peak to be 6.7 kDa, corresponding to the mass from 3FTxs previously recorded from *O. scutellatus*.

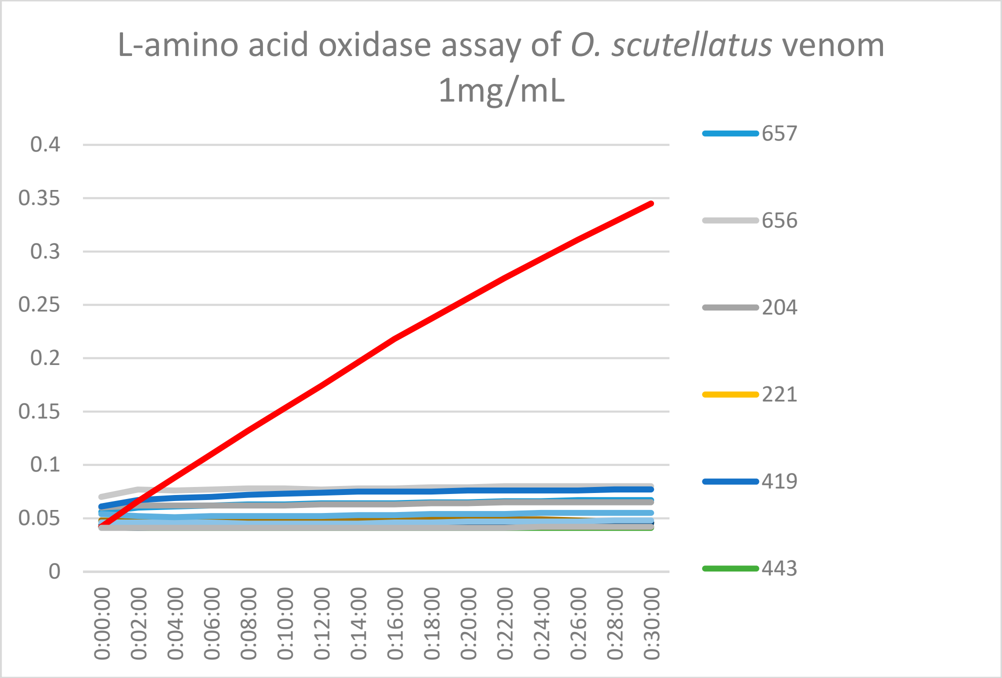

Figure S3. LAAO assay of all 13 taipans with venom of the elapid *Suta suta* as a positive control (red).
